# 244. Predictors and Outcomes of Multi-Drug Resistant Gram-Negative Bacteremia in Patients with Solid Tumours

**DOI:** 10.1093/ofid/ofad500.317

**Published:** 2023-11-27

**Authors:** George M Varghese, Bassem Awada, Jorge Abarca, Maniyando Milupi, Mansour Al Moundhri

**Affiliations:** Christian Medical College, Vellore, Tamil Nadu, India; Sultan Qaboos Comprehensive Cancer Care & Research Centre, Muscat, Masqat, Oman; Sultan Qaboos Comprehensive Cancer Care & Research Centre, Muscat, Masqat, Oman; Sultan Qaboos Comprehensive Cancer Care & Research Centre, Muscat, Masqat, Oman; Sultan Qaboos Comprehensive Cancer Care & Research Centre, Muscat, Masqat, Oman

## Abstract

**Background:**

Multi-drug resistant Gram-negative bacteraemia (MDR-GNB) is a major concern in cancer patients. However, predictors and outcomes of MDR-GNB bacteraemia in patients with solid tumours are not well defined. The objective of this study was to identify the predictors and outcomes of MDR-GNB bacteraemia in patients with solid tumours.

**Methods:**

This retrospective cohort study was conducted on patients with solid tumours admitted to a tertiary care hospital between January 2022 and December 2022. Demographic, clinical, and microbiological data were collected from electronic medical records. Univariate and multivariate analyses were performed to identify the predictors of MDR bacteraemia. The outcomes of interest included in-hospital 30-day mortality, length of hospital stay, and recurrence of bacteraemia.

**Results:**

Among 1074 patients with solid tumours, a total of 77 episodes of Gram-negative bacteremias occurred in 59 patients (47% male) with a mean age of 57.4 years. Of which 37(48%) had MDR-GNB bacteraemia. The most common source of bacteraemia was biliary (34.2%) followed by central line-associated (21%) and abdomen(21%). Carbapenem resistance was documented in 7(9.1%) of all Gram-negative bacteremias of which 75% were harbouring NDM with or without OXA-48 among enterobacteralis. The predictors of MDR-GNB bacteraemia were prior use of antibiotics (odds ratio [OR] 7.82, 95% [CI] 2.52,24.2) and *E.coli* bacteremia (OR 6.56, 95% CI 1.26,34.22). The median length of hospital stay was significantly longer in the MDR group compared to the non-MDR group (23 vs. 10.5 days; p= 0.003) and recurrence of bacteremia was significantly more common in the MDR group (35.13% vs. 5.0%; p=< 0.001). The 30-day in-hospital mortality was not significantly different (35.14% vs. 32.5%; p=0.81).

**Conclusion:**

This study identifies prior use of antibiotics and *E.coli* bacteremias as predictors of MDR-GNB bacteraemia in patients with solid tumours. Patients with MDR-GNB bacteraemia had longer hospital stays, and higher recurrence rates compared to those with non-MDR-GNB bacteremias. This highlights the importance of the judicious use of antibiotics and infection control measures to prevent MDR-GNB bacteraemia in this population.

**Disclosures:**

**All Authors**: No reported disclosures

